# Alterations and correlations of gut microbiota, fecal, and serum metabolome characteristics in a rat model of alcohol use disorder

**DOI:** 10.3389/fmicb.2022.1068825

**Published:** 2023-01-04

**Authors:** Xiaolong Wang, Lin Li, Cong Bian, Mingjian Bai, Haitao Yu, Han Gao, Jiaxin Zhao, Chunjing Zhang, Rongjie Zhao

**Affiliations:** ^1^Department of Medical Technology, Qiqihar Medical University, Qiqihar, Heilongjiang, China; ^2^Department of Psychiatry, Qiqihar Medical University, Qiqihar, Heilongjiang, China; ^3^National and Local United Engineering Laboratory for Chinese Herbal Medicine Breeding and Cultivation, School of Life Sciences, Jilin University, Changchun, China

**Keywords:** alcohol use disorder, gut microbiota, fecal metabolites, serum metabolites, alcohol preference

## Abstract

**Background:**

Growing evidence suggests the gut microbiota and metabolites in serum or fecal may play a key role in the process of alcohol use disorder (AUD). However, the correlations of gut microbiota and metabolites in both feces and serum in AUD subjects are not well understood.

**Methods:**

We established a rat model of AUD by a chronic intermittent ethanol voluntary drinking procedure, then the AUD syndromes, the gut microbiota, metabolomic profiling in feces and serum of the rats were examined, and correlations between gut microbiota and metabolites were analyzed.

**Results:**

Ethanol intake preference increased and maintained at a high level in experimental rats. Anxiety-like behaviors was observed by open field test and elevated plus maze test after ethanol withdraw, indicating that the AUD rat model was successfully developed. The full length 16S rRNA gene sequencing showed AUD significantly changed the β-diversity of gut microbial communities, and significantly decreased the microbial diversity but did not distinctly impact the microbial richness. Microbiota composition significantly changed in AUD rats, such as the abundance of *Romboutsia* and *Turicibacter* were significantly increased, whereas *uncultured_bacterium_o_Mollicutes_RF39* was decreased. In addition, the untargeted metabolome analysis revealed that many metabolites in both feces and serum were altered in the AUD rats, especially involved in sphingolipid metabolism and glycerophospholipid metabolism pathways. Finally, multiple correlations among AUD behavior, gut microbiota and co-changed metabolites were identified, and the metabolites were directly correlated with the gut microbiota and alcohol preference.

**Conclusion:**

The altered metabolites in feces and serum are important links between the gut microbiota dysbiosis and alcohol preference in AUD rats, and the altered gut microbiota and metabolites can be potentially new targets for treating AUD.

## Introduction

Drinking alcohol is associated with the risk of developing nearly 230 different types of diseases, such as mental and behavioral disorders, noncommunicable diseases such as liver cirrhosis, some cancers, and cardiovascular disease, as well as injuries resulting from violence and road collisions. Harmful use of alcohol is responsible for 5.1% of the global diseases burden. Alcohol consumption contributes to 3.3 million deaths annually and is the seventh leading risk factor for premature death and disability worldwide (Louisa Degenhardt et al., 2018; WHO, 2018, 2022). Alcohol-related morbidity and mortality are mainly due to the population’s high AUD prevalence. AUD, including alcohol abuse and dependence, based on the fifth edition of the Diagnostic and Statistical Manual of Mental Disorders (DSM), is characterized by a progressive escalation from low or moderate to excessive alcohol consumption, and by alcohol seeking and craving, and alcohol withdrawal symptoms such as anxiety, depression, restlessness, insomnia, tremor, seizures, which can be lethal (Ron and Barak, 2016; Fan et al., 2018; Xiao et al., 2018). AUD leads to disease burden and global adverse effects on social and economic factors. Thus, it is necessary and urgent to elaborate on AUD’s mechanisms and to develop more effective treatments.

Pathogenesis of AUD has been studied for decades, and neurobiological, genetic, environmental, psychosocial, gender, and age factors are some contributors to AUD development (Flores-Bonilla and Richardson, 2020; Guinle and Sinha, 2020; Egervari et al., 2021; Siomek-Gorecka et al., 2021). However, the molecular mechanisms underlying AUD have not been fully elucidated. The vast majority of researches have focused on the effect of alcohol consumption on the central nervous system in the brain and neuroendocrine system, such as the circuits in the prefrontal cortex (PFC), ventral tegmentum area (VTA), nucleus accumbens (NAc), and hypothalamic–pituitary–adrenal (HPA) axis (Goldstein and Volkow, 2002; Funk et al., 2006; Koob and Volkow, 2016). AUD formation was related to the alcohol’s toxicity effects on the multiple neurotransmitter systems, such as dopamine, gamma-aminobutyric acid, glutamate, serotonin, acetylcholine, and opioid systems (Yang et al., 2022). Therefore, many pharmacotherapies approved or currently under development are targeting at these neurotransmitter systems, but showed a limited effect (Burnette et al., 2022), which suggests that there are some other possible physiological processes play critical roles in the development of AUD (Leclercq et al., 2017; Fan et al., 2018). Studies found that the psychiatric disorders in AUD are also due to alcohol-induced dysregulation of the neuroimmune system (Erickson et al., 2019). Alcohol consumption and withdrawal cause neuroinflammation by leading to pro-inflammatory gene induction and microglial activation (Crews et al., 2021), which might be important for developing AUD and other psychological disorders (Robinson et al., 2014). In addition, AUD could increase intestinal permeability, allowing gut bacteria and gut-derived products to displace from the gut lumen to the systemic circulation or mesenteric lymph nodes (Leclercq et al., 2012, 2014a,b). These substances can activate the immune system, which then synthesize and release pro-inflammatory cytokines that reach the central nervous system and induce neuroinflammation associated with changes in mood, cognition, and drinking behavior (Leclercq et al., 2017). Compositional and functional changes in gut microbiota play a pivotal role in obesity and diabetes mellitus (Pitocco et al., 2020), cardiovascular disease (Witkowski et al., 2020), mental health (Dinan and Cryan, 2017), and alcoholic liver disease (Bajaj, 2019). Taken together, the gut microbiota and gut-derived bacterial products play a vital role in the AUD development.

Recently, several studies on the relationship between microbiota and AUD focused on determining the presence of dysbiosis in the gastrointestinal tract in AUD subjects in humans and rodent models (Vujkovic-Cvijin et al., 2020; Gupta et al., 2021) and the dysbiosis association with a variety of alcohol-related disorders, such as alcoholic liver disease (LeBrun et al., 2020) and mental disorders (Leclercq et al., 2020). There is increasing evidence that alcohol consumption can disturb the gut microbiota composition at different taxonomic levels. At the phylum level, alcohol consumption reduced the relative abundance of Bacteroidetes and increased Firmicutes (Addolorato et al., 2020; Smirnova et al., 2020). At the genus level, alcohol consumption increased the abundance of *Erysipelotrichia, Clostridium, Holdemania* and *Sutterella*, whereas decreased the abundance of *Allobaculum, Streptococcaceae, Enterobacteriaceae*, and *Faecalibacterium* (Bjørkhaug et al., 2019; Lang et al., 2020). However, some types of these gut microbiota changes also could be caused by other factors, such as obesity, a high-fat diet, and aging, demonstrating that this dysbiosis may not be specific to AUD (Ley et al., 2005; Hildebrandt et al., 2009; Mariat et al., 2009; Day and Kumamoto, 2022).

Gut bacteria can produce various bioactive metabolites, which can act locally in the gut or enter the host’s bloodstream *via* the portal vein (Fan and Pedersen, 2021), thereby affecting the fecal or serum metabolome. Besides the metabolites absorbed from the gut, serum metabolites, including nutrients, hormones, metabolic substrates, and products of cellular basal metabolism which can present some host cell basal metabolites. Compared to serum metabolites, fecal metabolites may be more representative of the direct microbial metabolic products produced in the gut. On the other hand, these metabolites eventually can enter the liver, muscle, adipose tissue, and brain, where they signal through targeting host molecules and affecting host signaling pathways (Koh and Bäckhed, 2020; Stavroulaki et al., 2022). While there have been many studies describe microbial metabolites in obesity, type 2 diabetes, non-alcoholic fatty liver disease, and other metabolic diseases (Pedersen et al., 2016; Wahlström et al., 2016; Federici, 2019). However, the microbial metabolites in AUD have been relatively unexplored.

It is now widely recognized that gut microbes play an important role in AUD development. In addition to the changes in gut microbiota composition, the metabolites produced by gut microbiota, including short-chain fatty acids (SCFAs), bile acid, secondary bile acid, serotonin, and taurine, also impact AUD (Postler and Ghosh, 2017; Wang et al., 2018; Yang et al., 2021). However, studies so far conducted only analyze the differences in fecal or serum metabolites during AUD and few reports integrate the relationship between differential metabolites in feces and serum. The combination of the two metabolomics approaches can provide a more comprehensive and detailed holistic metabolic profiling (Shan et al., 2018). The effect of alcohol consumption on both fecal and serum metabolites pathways in AUD is unclear. The associations among gut microbiota, gut metabolites, serum metabolites, and behavior changes in AUD are also unclear. Therefore, in the present study, the fecal microbiome, fecal and serum metabolomics profiles were characterized in a rat model of intermittent ethanol access induced AUD *via* full-length 16S rRNA sequencing and untargeted metabolomics analysis. The findings suggest that exploring the gut microbiome and related metabolites is extremely relevant for detecting new etiologies of AUD.

## Materials and methods

### Animals and experimental design

**Figure 1:**
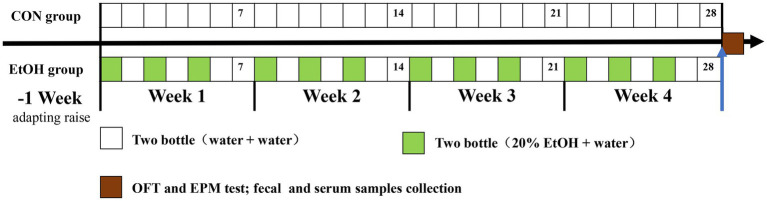
The schedule timeline of the IA2BC procedure in the experiment design. OFT represent open field test, EPM represent elevated plus maze test.

Adult male Sprague–Dawley rats (SPF grade; age: 8 weeks; weight: 240–290 g; *n* = 16) were obtained from Laboratory Animal Center in Qiqihar Medical University (Qiqihar, China). Animals were housed individually and kept under control conditions of 21–23°C, 40–60% humidity, and under a 12-hour regular light/dark cycle. Food and water were provided *ad libitum*. After 7 days of adapting to the environment, rats were randomly assigned to the experimental AUD model group by intermittent access to 20% ethanol in a 2-bottle choice (IA2BC) procedure as previously described by Carnicella (Carnicella et al., 2014; *n* = 8, weight = 263.67 ± 14.96 g, EtOH) and a control group (*n* = 8, weight = 264.33 ± 23.25 g, CON). Briefly, the EtOH group rats received free 24-h drinking of 2 bottles of selected wine (containing water and 20% ethanol) three times per week (usually Monday, Wednesday, and Friday), with 24-h and 48-h withdrawal periods on weekdays and weekends, respectively. During ethanol withdrawal, rats received two bottles of water. The placement of ethanol bottles was alternated during each drinking session to control side preferences (Carnicella et al., 2014). Rats in the CON group received free 24-h drinking of 2 bottles of water. Liquid consumption, food intake, and body weight were determined every day. After 4 weeks of experimental AUD model establishment, an open field test and an elevated plus maze test were performed to evaluate the anxiety level of each rat. The experimental schedule was showed in Figure 1. All procedures were approved by the Animal Ethical Care Committee of Qiqihar Medical University and followed ‘Guidelines for the Care and Use of Laboratory Animals’ published by the Chinese Animal Welfare Committee.

### Anxiety-like behavior measurements

#### Open field test

The open field test (OFT) is commonly used to assay anxiety-like behaviors, and the decrease in distance and time in the center field indicates increased anxiety. The apparatus consisted of a square box that measured 43 × 43 cm with 45 cm walls, and the test arena was divided into central (15 × 15 cm) and peripheral zones. The apparatus was placed under a homogenous illumination (14–20 lx). The methods according the references of Cheng (Cheng et al., 2020) with some modifications. Each rat was gently placed in the central area and was allowed to explore for 5 min. The time spent in the center, the distance traveled in the center as a percentage of the total distance, the total distance traveled, and resting time was recorded and analyzed by an automated video tracking system (ZhongShiKeJi Co., Beijing, China). Between each subject, the field was wiped clean to avoid cue smell.

#### Elevated plus maze

In addition, the elevated plus maze (EPM) is one of the most widely used methods for measuring anxiety-like behaviors. The facility is a plus-shaped maze composed of two open arms (width, 10.00 cm, length, 50.00 cm) and two closed arms (width, 10.00 cm, length, 50.00 cm, walls, 30.00 cm) with a central platform (10.00 cm × 10.00 cm). The EPM was 80 cm elevated from the floor. The methods according the references of Jiao (Jiao et al., 2021) with some modifications. Rats were placed on the central platform facing an open arm individually, and the activity of the rat in the maze for 5 min was recorded by an automated video tracking system. The maze was wiped clean after each test. The time spent in the open arms as a percent of the total time spent exploring the open and closed arms (percentage of time spent in the open arm) and the number of entries into open arms as a percentage of the total number of entries into both open and closed arms (percentage of entries into open arms) were used as an index of anxiety. Anxiety-like behavior is associated with decreased time and entries in the open arms.

### Sample collection

After 4 weeks of AUD model establishment, fecal samples were collected (*n* = 8 per group) and placed in two sterile plastic tubes from each rat for microbial and metabolomic analysis, respectively. Next, the samples were rapidly snap-frozen in liquid nitrogen and stored at −80°C until used. Finally, blood was collected from the heart under isoflurane anesthesia. Then the blood was centrifuged to separate the serum and stored frozen at −80°C for the following metabolic analysis.

### Full length 16S rRNA gene sequencing

Total genome DNA of fecal samples was extracted with QIAamp Power Fecal DNA Kit (QIAGEN, Germany) following the manufacturer’s protocol. DNA quantity and quality were assessed using an ultramicro-spectrophotometer B-500 (Shanghai Metash Instruments Co., Ltd., China). The bacterial communities in the fecal samples were investigated by PacBio Sequel (Biomarker Technologies Company, Beijing, China). The full-length 16S rRNA genes were selected for PCR with the barcoded primers 27F (5′-AGRGTTYGATYMTGGCTCAG-3′) and 1492R (5′-RGYTACCTTGTTACGACTT-3′), where the barcode was an eight base sequence unique to each sample. PCRs were performed according to the following volume: 25 μl reactions mixtures containing 5 μl of 5× FastPfu buffer, 5 μM each primer, 2 μl of 2.5 μM dNTPs, 0.4 μl FastPfu Polymerase, 10 ng template DNA, and ddH_2_O making up to 25 μl. The cycling program was as follows: denaturation at 95°C for 3 min, 25 cycles (95°C for 30 s, 55°C for 30 s, and 72°C for 45 s), and a final extension at 72°C for 10 min. The PCR products were checked on a 1.5% agarose gel and further purified, then pooled in equimolar and sequenced on PacBio Sequel (Pacific Biosciences, USA).

The circular consensus sequences (CCS) were obtained by SMRT Link v 8.0 software with the index set as min Passes ≥5, min Predicted Accuracy≥0.9. For further sequence processing, Lima v1.7.0 and Cutadapt v2.7 were used to recognize the CCS by barcode and filter the length of 1,200 bp-1,650 bp CCS, respectively. Further details of the sequencing output, such as the average length, error rate, and quality, are summarized in Supplementary Table S1. Operational taxonomic units (OTUs) were clustered with a 97% similarity cutoff using USEARCH v10.0, and chimeric sequences were identified and removed using UCHIME v4.2. The taxonomy of each OTUs was performed using classify. Seqs by the RDP classifier implemented in Silva v132 16S rRNA database using a confidence threshold of 70% (Callahan et al., 2019). The alpha-diversity indices, including Chao, ACE, Shannon, and Simpson, were calculated to estimate microbiota diversity and abundance in each sample using QIIME 2 software. Beta-diversity indices were analyzed using principal co-ordinates analysis (PCoA), ANOSIM analysis and heatmap to show the composition of the gut microbiota communities in the different gut segment samples, and an analysis of molecular variance (AMOVA) was performed to compare the difference between EtOH and CON group.

### Untargeted metabolomics analysis

#### Sample preparation

Fecal samples (50 mg) were used and thawed on ice. To these samples 1,000 μl of extraction liquid containing an internal target (1,000:2; V methanol:V acetonitrile:V water = 2:2:1) was added, and vortex for 30 s. Then the samples were homogenized in a bead mill for 10 min at 45 Hz and ultrasonicated for 10 min (in an ice water bath). The samples were centrifuged at 15,000 g for 15 min at 4°C after incubating for 1 h at −20°C. Then 500 μl of the supernatant was dried in a vacuum concentrator without heating. After 160 μl of extraction liquid (V acetonitrile:V water = 1:1) was added for reconstitution. The samples were vortexed for 30 s, sonicated for 10 min (in an ice water bath), and centrifuged at 15000 g for 15 min at 4°C. Finally, the supernatant was carefully transferred (120 μl) to a fresh glass vial and stored at −80°C until metabolomics analysis.

For serum samples, 100 μl serum was used and thawed on ice, and after 300 μl of cold acetonitrile was added and the samples vortex for 30 s and incubated for 1 h at −20°C. Then the samples were centrifuged at 15,000 g for 15 min at 4°C. The supernatant was collected and dried under a stream of nitrogen. Next, the samples were reconstituted in 1:1 water/acetonitrile, vortexed for 30 s, and kept at 4°C for 20 min. Then samples were centrifuged at 15,000 g for 15 min at 4°C. Finally, the supernatant was carefully transferred to fresh vials for Liquid Chromatography-Mass Spectrometry (LC/MS) analysis. The quality control (QC) sample was mixed with 10 μl of each sample.

#### UHPLC-QTOF-MS analysis

The UHPLC-QTOF-MS system for metabolomic analysis is composed of Waters Acquity I-Class PLUS ultra-high performance liquid chromatography with Acquity UPLC HSS T3 column (1.8 μm 2.1*100 mm, Waters) coupled to a Xevo G2-XS QTof high-resolution mass spectrometer. The mobile phase consisted of 0.1% formic acid aqueous solution (A) and 0.1% formic acid acetonitrile (B) for positive ion mode, was performed with elution gradient as follows: 0 min, 98% A; 0.25 min, 98% A; 10.0 min, 2% A; 13 min, 2% A; 13.1 min, 98% A; and 15 min, 98% A at 400 μl/min. The injection volume used in this study was 1 μl. The Waters Xevo G2-XS QTOF high-resolution mass spectrometer can collect primary and secondary mass spectrometry data in MSe mode under the control of the acquisition software (MassLynx V4.2, Waters). In each data acquisition cycle, dual-channel data acquisition can be performed on both low collision energy and high collision energy at the same time. The low collision energy was 2 V; the high collision energy range was 10–40 V; and the scanning frequency was 0.2 s for a mass spectrum. The parameters of the electron spray ionization (ESI) ion source were as follows: capillary voltage: 2,000 V (positive ion mode) or − 1,500 V (negative ion mode); cone voltage: 30 V; ion source temperature: 150°C; desolvent gas temperature 500°C; backflush gas flow rate: 50 L/h; desolventizing gas flow rate: 800 L/h.

### Data preprocessing, annotation, and analysis

The raw data was collected using MassLynx V4.2 and was processed by Progenesis QI software for peak extraction, peak alignment, and other data processing operations, based on the Progenesis QI software online METLIN database and Biomark’s self-built library for identification. At the same time, the theoretical fragment identification and mass deviation were within 100 ppm. After normalizing the original peak area information with the total peak area, a follow-up analysis was performed. The identified compounds were searched for classification and pathway information in the databases of KEGG, HMDB, and lipid maps. T-test was used to calculate the significant differences (*p* value) of each compound between EtOH and CON groups. The unsupervised principal component analysis (PCA) and supervised orthogonal partial least squares discriminant analysis (OPLS-DA) was performed by R packages to characterize metabolic perturbation and differences between groups. The variable important in projection (VIP) value of the model was calculated using multiple cross-validations. The method of combining the difference multiple, the *p* value, and the VIP value of the OPLS-DA model was adopted to screen the differential metabolites. The screening criteria are FC > 2 or FC < 0.5, *p* value <0.05 and VIP >1. We plotted the results on a volcano map. The different metabolites of KEGG pathway enrichment significance were performed by the Web-based tool MetaboAnalyst 5.0^1^. The correlations between major differences between gut microbiota and metabolites were performed by Spearman’s correlation coefficients analysis in the R package.

## Results

### AUD symptoms seen in the rats

**Figure 2:**
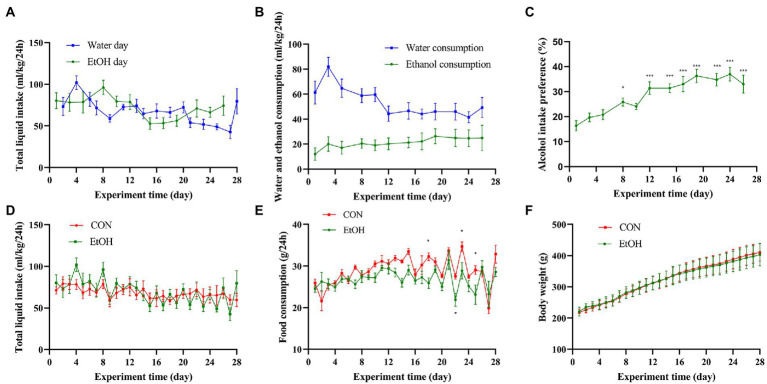
IA2BC affected alcohol intake preference but did not affect body weight or liquid/food intake. **(A)** The total liquid intake showed no significant difference between the water day and EtOH day in EtOH group. **(B)** The ethanol consumption was increasing with the water consumption was decreasing in IA2BC rats. **(C)** Alcohol intake preference was increasing with chronic intermittent ethanol voluntary drinking time. The total liquid intake **(D)**, food consumption **(E)** or body weight **(F)** did not altered by chronic intermittent ethanol voluntary drinking. Data are presented as Mean ± SEM, *n* = 8/group; compared with the first day, *p < 0.05, **p < 0.01, ***p < 0.001.

During the 4 weeks experimental procedure, IA2BC procedure successfully induced AUD symptoms in SD rats. In the EtOH group, there was no significant differences were detected in the total liquid intake between the water day and EtOH day (Figure 2A), the percentage of alcohol intake was increasing whereas the percentage of water intake was decreasing (Figure 2B). On the other hand, the alcohol intake preference increased with the experimental time, then maintained at a high level, and it was significantly higher after the 12th day than that on the first day (Figure 2C). Additionally, no significant difference was observed between the EtOH and CON group in the total liquid intake, food consumption and body weight (Figures 2D-F).

**Figure 3:**
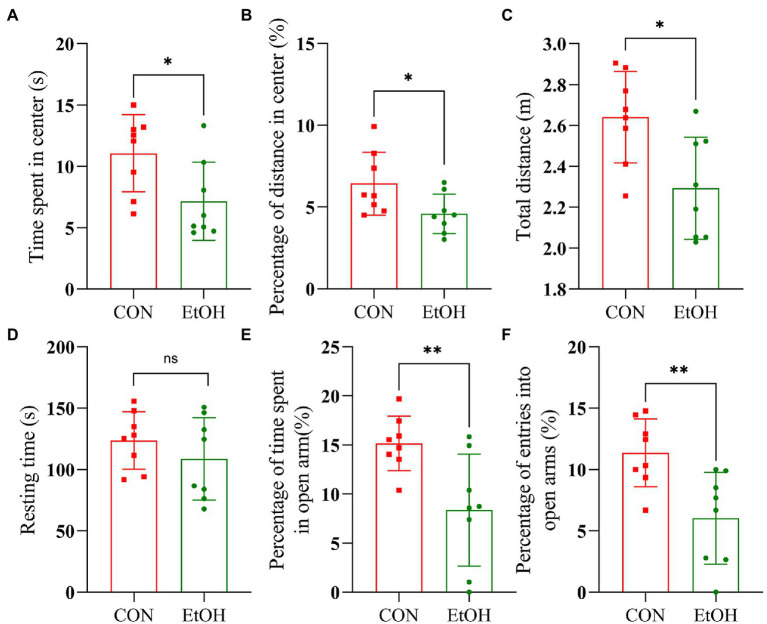
IA2BC rats exhibited anxiety-like behaviors. Behavior test was performed at the 28th day of the procedure. In the open field test, EtOH group rats spent less time in the center zone (*t* = 2.47, *p* = 0.027), traveled significantly less distance in the center zone (*t* = 2.30, *p* = 0.038) and less total distances (*t* = 2.94, *p* = 0.011) than that of CON group **(A–C)**. The resting time between EtOH and CON group had no significant difference (*t* = 1.04, *p* = 0.351, **D**). In the elevated plus maze, the EtOH group spent less time in open arms (*t* = 3.04, *p* = 0.009) and the number entries into open arms significantly reduced (*t* = 3.24, *p* = 0.006) compared with CON group **(E,F)**. Results are displayed as means ± SD. Significant results were determined by unpaired *t*-tests, *p < 0.05, **p < 0.01.

AUD is often accompanied by mental disorders such as anxiety. Therefore, to confirm the establishment of AUD experimental animal models, open field test (OFT) and elevated plus maze (EPM) tests were performed at the end of the experimental procedure. In the OFT, anxiety-like behaviors were observed in this experimental group. In detail, the time spent in the center zone (*t* = 2.47, *p* = 0.027), the distance traveled in the center zone (*t* = 2.30, *p* = 0.038), and the total distance (*t* = 2.94, *p* = 0.011) was significantly reduced in the EtOH group compared to the CON group (Figures 3A-C), while the resting time was similar (Figure 3D). Additionally, the number of entries into open arms and the time spent in the open arms of the EPM was significantly decreased in the EtOH group compared to the CON group (Figures 3E,F), indicating an increase in the anxiety-like behavior in the rats of the EtOH group. The alcohol intake preference and anxiety-like behavior indicate that the AUD rat model was successfully developed in EtOH group.

### Fecal microbiota changed in AUD rats

In total, 195,848 circular consensus sequencing (CCS) was obtained after barcode recognition, and 176,494 effective CCS were obtained after quality control and chimera filtering. On average, 10,722 and 11,340 effective CCS per sample were obtained from the EtOH and CON groups fecal contents, respectively, with an average length of 1,456 bp. These sequences were assigned to 641 bacterial OTUs, including 17 phylum, 23 classes, 44 orders, 64 families, 131 genera, and 157 species. In this study, 590 OTUs were shared, and 36 and 15 were obtained from the CON and EtOH groups, respectively (Supplementary Figure S1).

**Figure 4:**
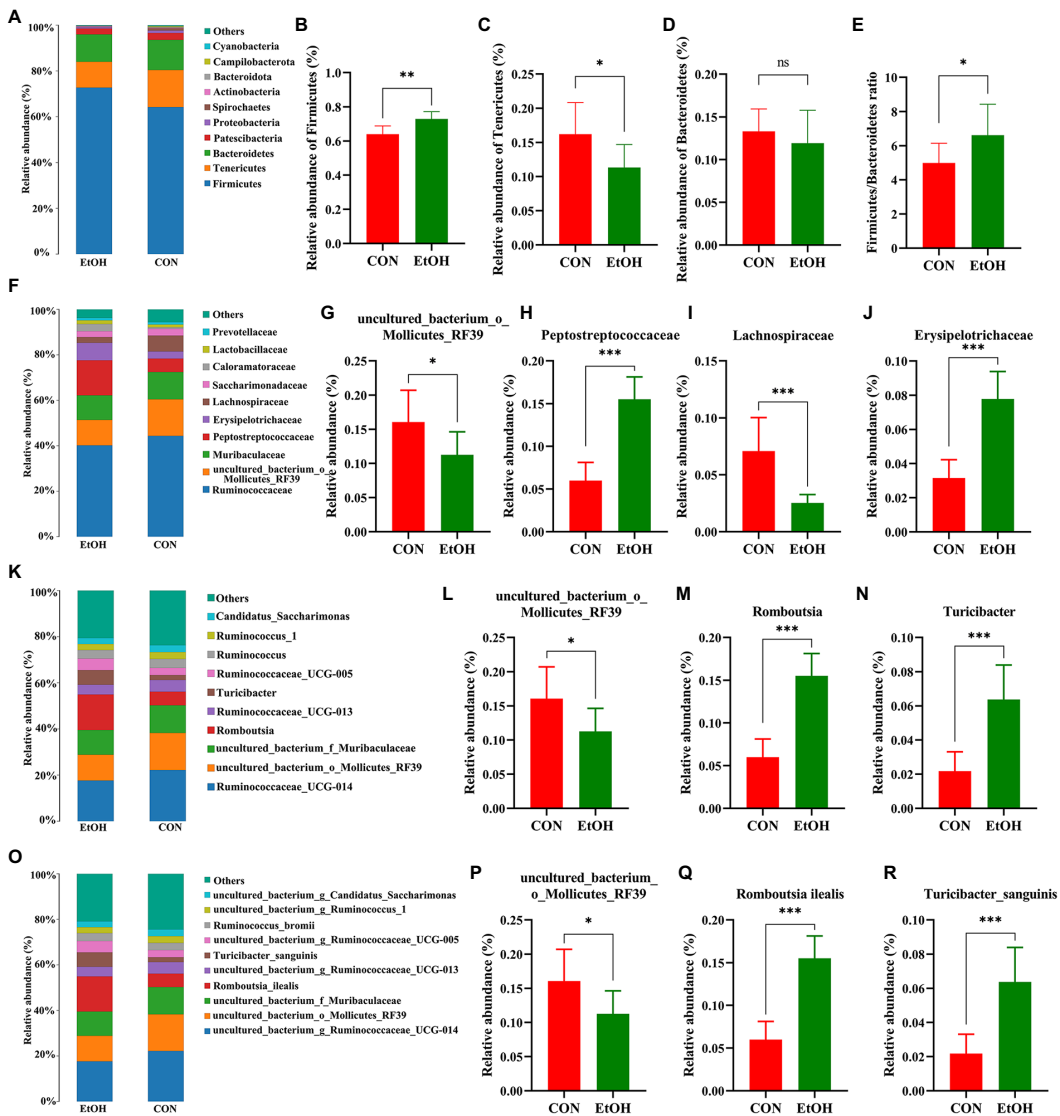
Chronic intermittent ethanol voluntary drinking alters the composition of gut microbiota in rats. Histogram of top 10 relative abundance at different level. The relative abundance and the main bacterial genera between the two groups with Wilcoxon rank-sum test at the phylum **(A–E)**, family **(F–J)**, genus **(K–N)**, and species **(O–R)** level in the fecal samples. Values are presented as the mean ± SD (*n* = 8 per group), *p < 0.05, **p < 0.01, ***p < 0.001.

The distribution of bacterial taxon and the top 10 relative abundance of bacteria at the different taxon levels are shown in Figure 4. At the level of phylum taxon (Figures 4A–E), Firmicutes, Tenericutes, and Bacteroidetes were the most abundant phylum in the two groups accounting for 95.95% of relative abundance in the EtOH group and 93.30% in the CON group. The relative abundance of Firmicutes in the EtOH group (72.91 ± 1.49%) was significantly higher than that in the CON group (64.00 ± 1.71%; *t* = 3.93, df = 14, *p* < 0.01).

The relative abundance of Tenericutes in the EtOH group (11.32 ± 1.20%) was significantly lower than that in the CON group (16.20 ± 1.64%; *t* = 2.41, df = 14, *p* = 0.03). Additionally, the relative abundances of Bacteroidetes in the EtOH and CON group were 11.94 ± 1.36% and 13.32 ± 0.92%, respectively (no statistically significant differences were detected: *t* = 0.84, df = 14, *p* = 0.41). The ratio of Firmicutes to Bacteroidetes was significantly higher in the EtOH group than that in the CON group.

At the family level (Figures 4F–J), Ruminococcaceae exhibited the highest proportion in the EtOH group (40.10 ± 3.71%) and CON group (43.99% ± 6.81%), with no significant differences between groups (*t* = 1.42, df = 14, *p* = 0.18). Moreover, the relative abundance of uncultured_bacterium_o_Mollicutes_RF39 and Lachnospiraceae was significantly lower, whereas Peptostreptococcaceae and Erysipelotrichaceae were significantly higher in the EtOH group than that in the CON group. Furthermore, a decrease in the abundance of *uncultured_bacterium_o_Mollicutes_RF39* was observed at the genus level. On the other hand, an increase in the abundance of *Romboutsia* and *Turicibacter* was observed in the EtOH group compared to the CON group (Figures 4K–N). At the species level (Figures 4O–R), the AUD significantly decreased the abundance of *uncul-tured_bacterium_o_Mollicutes_RF39* and significantly increased the abundance of *Romboutsia ilealis* and *Turicibacter sanguinis* in the EtOH group compared to the CON group.

**Figure 5:**
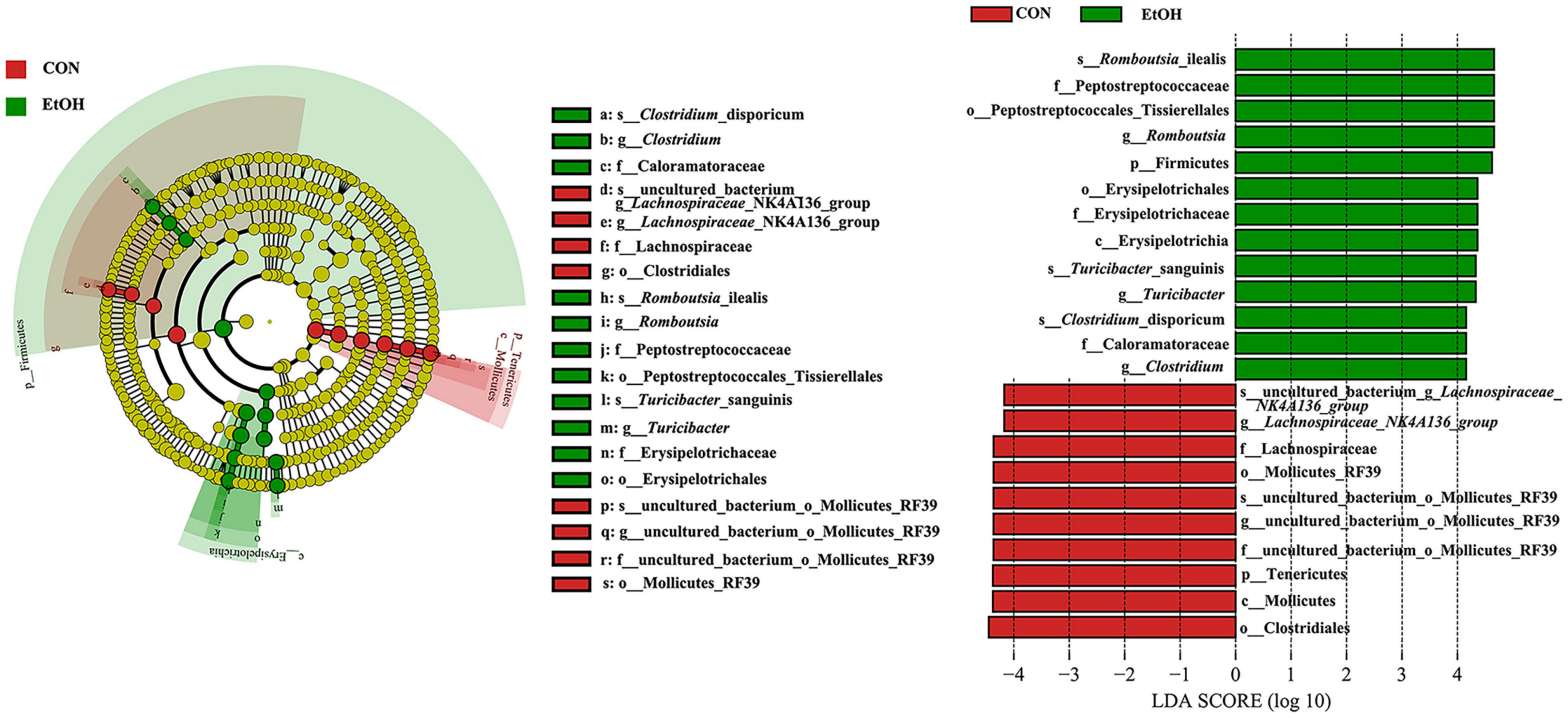
Linear discriminant analysis Effect Size (LEfSe) analysis between the EtOH and CON groups, with a LAD score > 4.

A linear discriminant analysis effect size (LEfSe) analysis was performed according to linear discriminant analysis (LDA) fold = 4, and the relationship between different microbiota from the phylum to the species levels is shown in the cladogram (Figure 5). The results showed 23 OTUs at the phylum (2 OTUs), class (2 OTUs), order (4 OTUs), family (5 OTUs), genus (5 OTUs), and species levels (5 OTUs) were significantly different between the EtOH and CON groups. The relative abundances of 13 OTUs were higher in the EtOH group. However, 10 OTUs were more abundant in the CON group. Among the significantly differential OTUs, at the species level, *Clostridium_disporicum*, *Romboutsia_ilealis*, and *Turicibacter_sanguinis* were the three most abundant bacteria in the EtOH group, while *uncultured_bacterium_g_Lachnospiraceae_NK4A136_group* and *uncul-tured_bacterium_o_Mollicutes_RF39* were the most abundant in the CON group.

**Figure 6:**
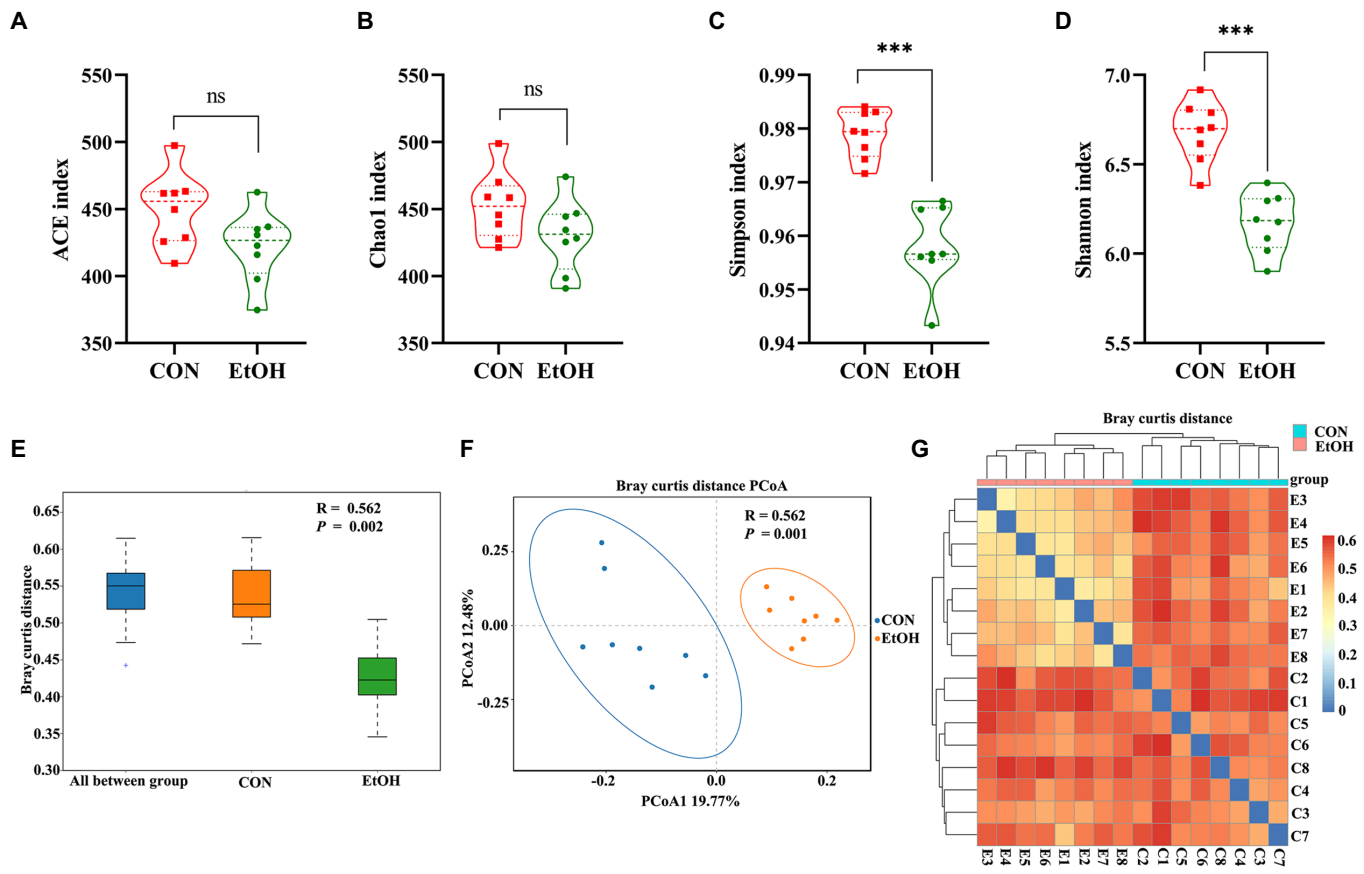
Chronic intermittent ethanol voluntary drinking alters the diversity of gut microbiota in rats. Difference in alpha diversity index between EtOH and CON group showed in the plot **(A–D)**. **(A)** represent ACE index; **(B)** represent Chao1 index; **(C)** respresent Simpson index; **(D)** represent Shannon index. The width of each curve in the violin plot corresponds with the approximate frequency of data points in each region, dotted line indicating the median value and quartile positions. Significant results were determined by unpaired *t*-tests, ***p < 0.001; ns, *p* > 0.05. **(E)**, ANOSIM analysis showed differences between EtOH and CON groups were significantly greater than those within groups. **(F)** PCoA plot based on Bray–Curtis distance, EtOH and CON groups could be effectively separated, showing that the composition of the gut microbiota in EtOH group was significantly different from that of CON group. **(G)** samples heat map based on the Bray–Curtis distance showed that the samples in the same group clustered together, indicating that EtOH changed the gut microbiota community.

The chao1 richness and the abundance-based coverage estimator (ACE) richness indices were slightly lower in the EtOH group than in the CON group, but their differences were not statistically significant (Figures 6A,B). The obtained richness and diversity indices of the fecal microbiota showed that AUD significantly decreased the Shannon and Simpson diversity indices (Figures 6C,D). The results suggested that AUD significantly impacted the microbial diversity but did not distinctly impact the fecal microbial richness in the rats. Furthermore, using β-diversity analysis of ANOSIM based on Bray–Curtis distance, vastly different bacterial community structures were observed between the two groups (*R* = 0.562, *p* = 0.002; Figure 6E). The PCoA of Bray–Curtis distance performed on the OTU abundance matrix showed that PCoA1 explained 19.77% of variance and PCoA2 explained 12.48% of the variance (*R* = 0.562, *p* = 0.001). All the samples in the same group were grouped into one cluster (Figure 6F). Moreover, comparing heatmaps based on Bray–Curtis distance confirmed these results. A lower distance was observed among the samples in the EtOH group, and the samples in the same group clustered together (Figure 6G). The results showed that AUD significantly changed the β-diversity of gut microbial communities in rats.

### The fecal metabolic profile changed in AUD rats

**Figure 7:**
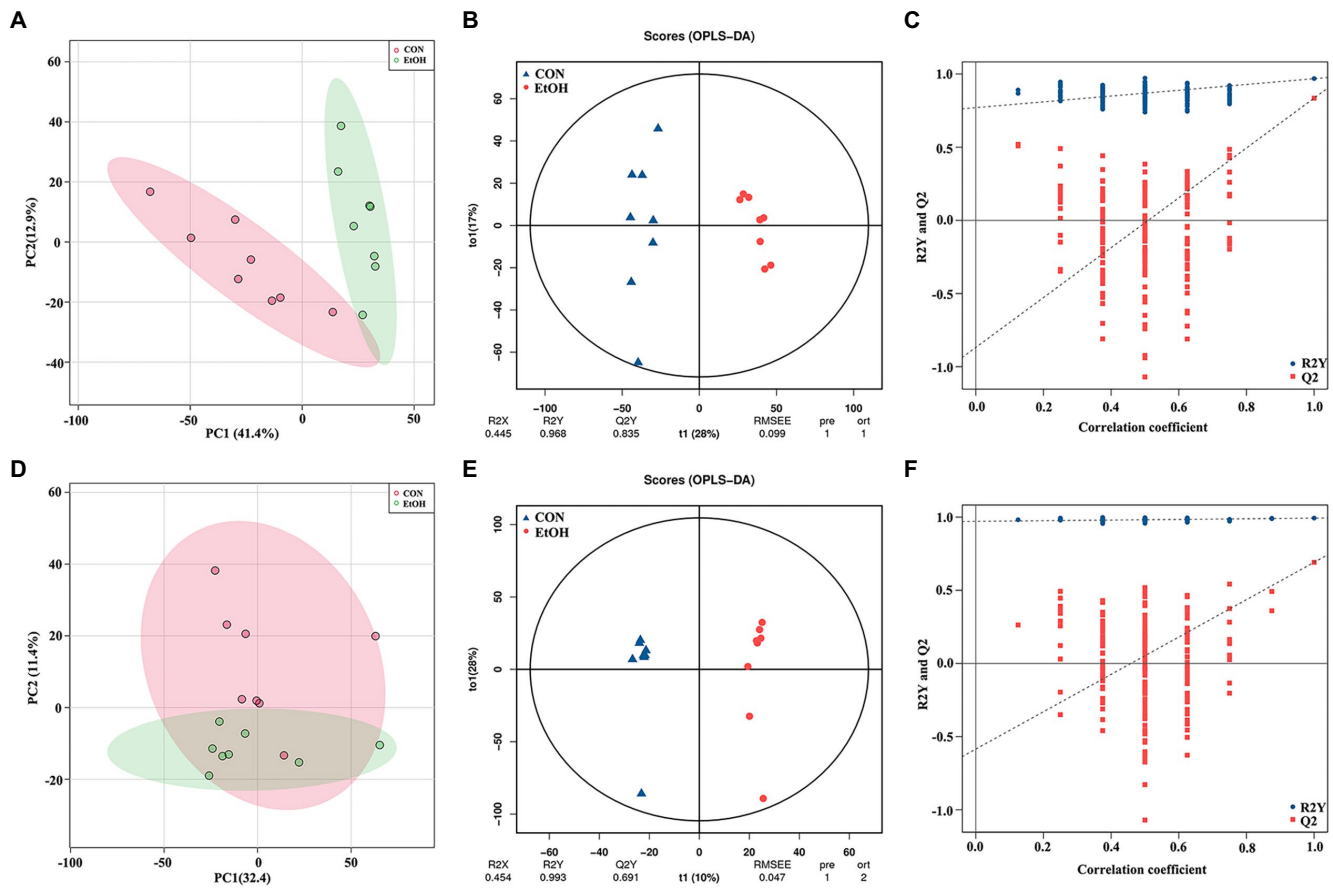
Multivariate statistical analysis of fecal metabolites measured by untargeted metabolomics analysis at positive **(A–C)** and negative **(D–F)** ion mode. PCA analyses comparing metabolites of all samples between EtOH and CON group **(A,D)**; OPLS-DA scores showed significant differences between EtOH and CON group **(B,E)**; and the OPLS-DA permutation test confirmed the differences of fecal metabolites in EtOH and CON group **(C,F)**.

**Figure 8:**
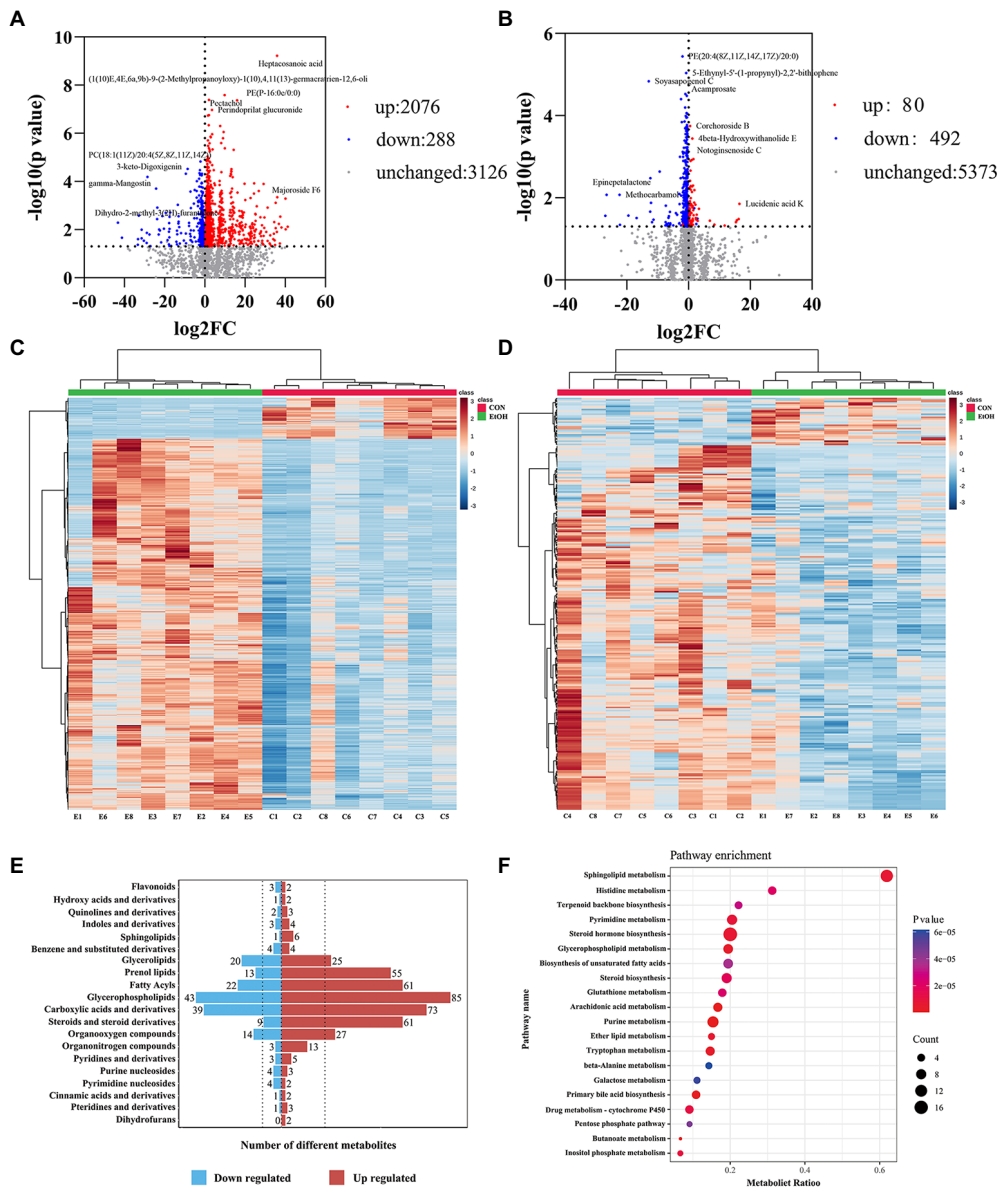
The fecal metabolic profile changed in AUD rats. Differential metabolites in fecal identification between EtOH and CON group at positive **(A,C)** and negative **(B,D)** ion mode. Expression of differential metabolites in the two groups was represented by Volcano plot **(A,B)** and heat maps **(C,D)**. The number of differential metabolites were annotated and classified based on the HMDB database **(E)**. The enrichment pathway of fecal differential metabolites by Kyoto encyclopedia of genes and genomes (KEGG) analysis **(F)**.

Untargeted liquid chromatography mass spectrometry (LC–MS) was used to detect the fecal metabolites in the EtOH and CON groups. In total, 5,490 metabolites in positive ionization mode and 5,945 metabolites in negative ionization mode were detected. To investigate the fecal metabolites differences between EtOH and CON rats, PCA and OPLS-DA were performed (Figure 7). The PCA results showed that the EtOH rats were clearly distinguished from the CON rats. Furthermore, the OPLS-DA results revealed that fecal metabolites significantly differed between EtOH and CON rats. The OPLS-DA permutation test validated the model, and the results demonstrated that the fecal metabolites were changed in EtOH compared to the CON rats, suggesting that AUD changed the fecal metabolome composition. Moreover, metabolites with VIP > 1 and *p* < 0.05 were considered to be significantly altered by AUD (Supplementary Data S1, S2). In the positive ionization mode, 2076 metabolites were up regulated, and 288 metabolites were down regulated. On the other hand, in the negative ionization mode, 80 metabolites were up regulated, and 492 metabolites were down regulated in the EtOH group compared to the CON group, showed in the volcano plot and heatmap in Figures 8A–D. The differential metabolites were annotated and classified based on the HMDB database. The results showed that most of the differential metabolites in fecal belonged to glycerophospholipids, carboxylic acids and derivatives, fatty acyls (Figure 8E), and were classified as amino acids, peptides and analogs, glycer-ophosphoethanolamines, fatty acids and conjugates, glycerophosphocholines, and diradylglycerols in the subclass level (Supplementary Figure S2). The concentrations of cortisone, glutathione, sphingosine, and L-tyrosine were significantly increased in EtOH rats, whereas butyric acid, dopamine, L-glutamine, and L-aspartic acid were decreased (Supplementary Figure S3). The equations should be inserted in editable format from the equation editor.

To further explore the pathways associated with these altered metabolites by AUD, KEGG pathway enrichment analyses were performed by MetaboAnalyst 5.0. The top 20 significant pathways are shown in Figure 8F. We found that the lipid metabolism, amino acids metabolism, nucleotide metabolism, and carbohydrate metabolism pathways were involved in the process of the AUD, including sphingolipid metabolism, glycerophospholipid metabolism, steroid hormone biosynthesis, histidine metabolism, tryptophan metabolism, and pentose phosphate pathway. The results suggested that AUD altered the fecal metabolic profile in rats.

### Correlations of the fecal metabolites and gut microbiota

**Figure 9:**
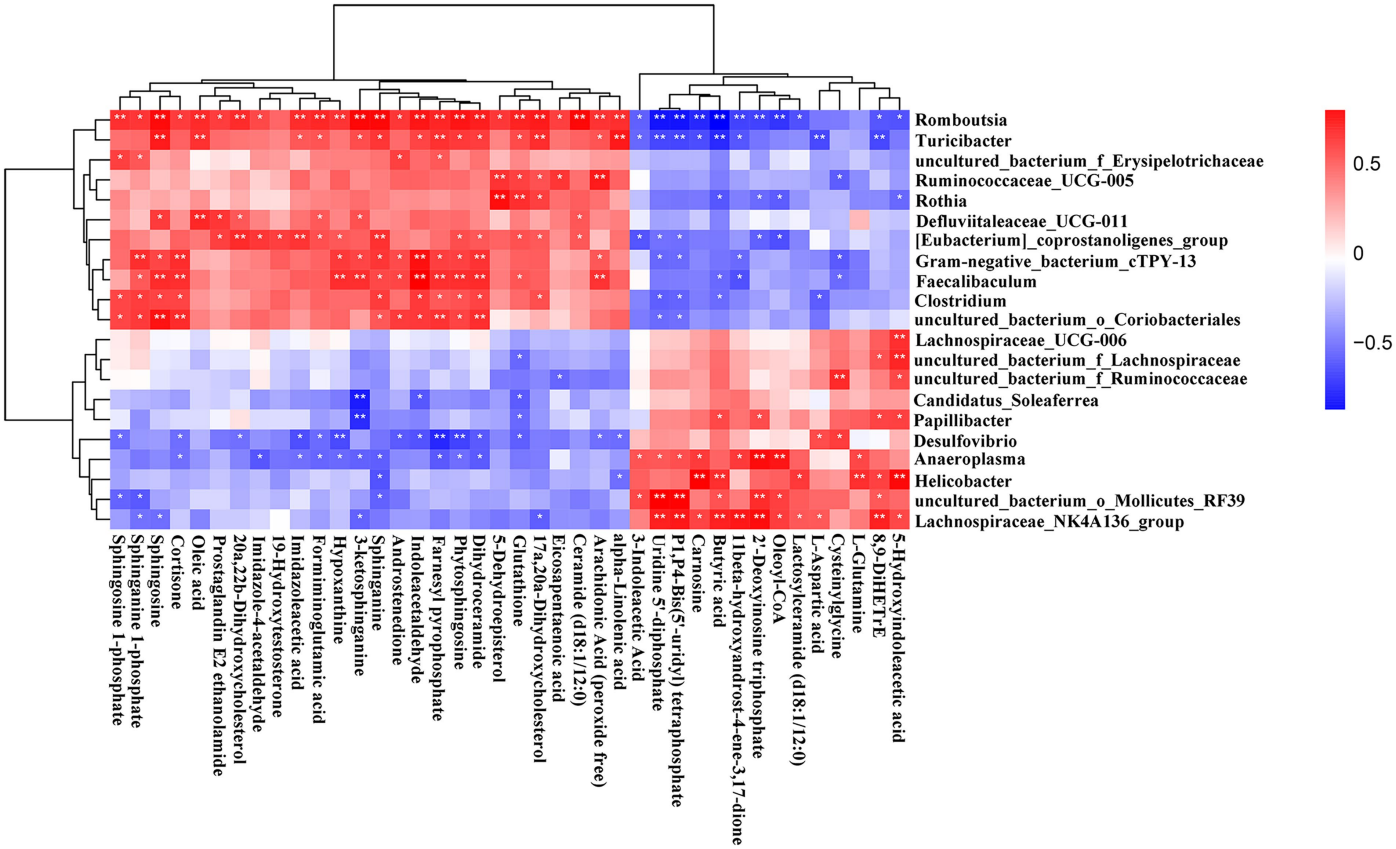
Correlation heatmap of differential microbiota and fecal metabolites. Data were calculated by Spearman’s correlation method after mean centering and unit variance scaling. *p < 0.05, ** *p* < 0.01.

To explore the functional correlations between the fecal metabolites and the gut microbiota, Spearman’s correlation analysis was performed. A strong correlation was found between the top 40 significantly enriched in the KEGG pathway metabolites and the discriminated gut microbiota at the genus level (Figure 9). *Romboutsia* and *Turicibacter* were positively correlated with sphingosine, oleic acid, farnesyl pyrophosphate, 17α, 20α-dihydroxycholesterol, and alpha−linolenic acid but negatively correlated with uridine 5′-diphosphate, P1,P4-bis(5- uridyl) tetraphosphate, and butyric acid. Similarly, *Gram − negative_bacterium_cTPY-13*, *Faecalibaculum*, *Clostridium*, and *uncul-tured_bacterium_o_Coriobacteriales* were positively correlated with sphinganine 1-phosphate, sphingosine, cortisone, sphinganine, indoleacetaldehyde, farnesyl pyrophosphate, phytosphingosine, and dihydroceramide. However, *Lachnospirace-ae_NK4A136_group* and *uncultured_bacterium_o_Mollicutes_RF39* were positively correlated with uridine 5′-diphosphate, P1,P4-bis(5′-uridyl) tetraphosphate, butyric acid, 2′-deoxyinosine triphosphate, oleoyl-CoA, and 8,9-DiHETrE. *Helicobacter* was positively correlated with carnosine, butyric acid, L-glutamine, and 5-hydroxyindoleacetic acid. On the other hand, Anaeroplasma was positively correlated with 2′-deoxyinosine triphosphate and oleoyl-CoA. Moreover, *Lachnospiraceae_UCG-006* and *uncul-tured_bacterium_f_Lachnospiraceae* were positively correlated with 5-hydroxyindoleacetic acid, and *uncultured_bacterium_f_Ruminococcaceae* was positively correlated with cysteinylglycine. Additionally, *Desulfovibrio* was negatively correlated with hypoxanthine, farnesyl pyrophosphate, and phytosphingosine. Finally, *Candidatus_Soleaferrea* and *Papillibacter* were negatively correlated with 3-ketosphinganine. The correlation results showed that AUD could significantly change the gut leading to significant changes in the fecal metabolites.

### The serum metabolic profile changed in AUD rats

**Figure 10:**
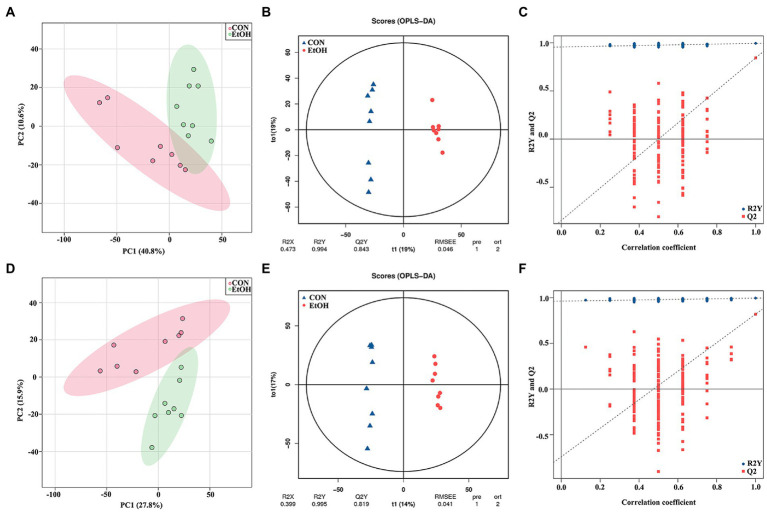
Multivariate statistical analysis of serum metabolites measured by untargeted metabolomics analysis at positive **(A–C)** and negative **(D–F)** ion mode. PCA analyses comparing metabolites of all samples between EtOH and CON group **(A,D)**; OPLS-DA scores showed significant differences between EtOH and CON group **(B,E)**; and the OPLS-DA permutation test confirmed the differences of serum metabolites in EtOH and CON group **(C,F)**.

**Figure 11:**
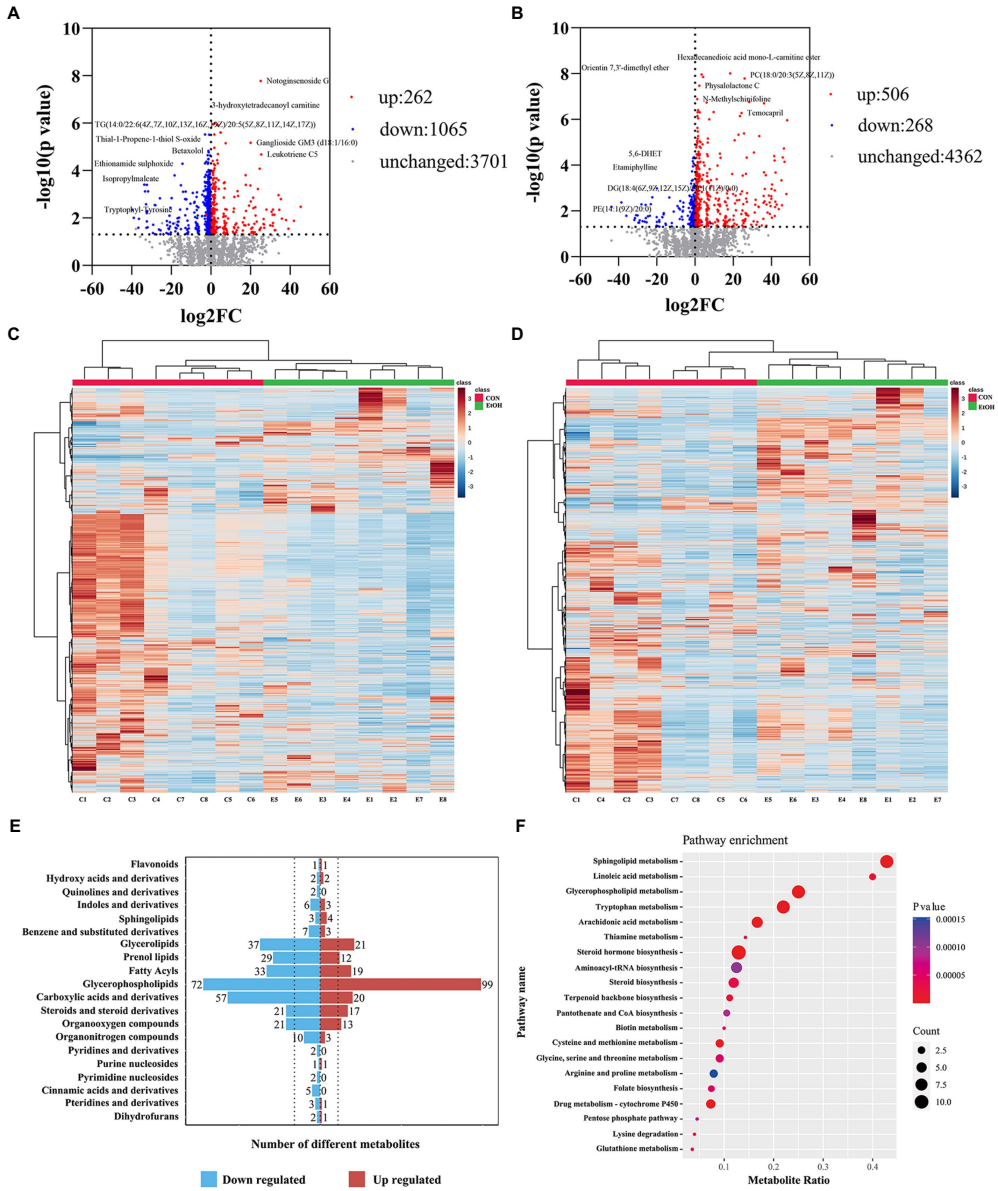
Differential metabolites in serum identification between EtOH and CON group at positive **(A,C)** and negative **(B,D)** ion mode. Expression of differential metabolites in the two groups was represented by Volcano plot **(A,B)** and heat maps **(C,D)**. The number of differential metabolites were annotated and classified based on the HMDB database **(E)**. The enrichment pathway of serum differential metabolites by Kyoto encyclopedia of genes and genomes (KEGG) analysis **(F)**.

To identify the differences in the serum metabolites in AUD rats, untargeted LC–MS for metabolomics analysis of serum was used in EtOH and CON rats. In total, 5,028 and 5,136 metabolites were detected in the positive and negative ionization modes, respectively. The PCA analysis showed significant differences in the metabolite profiles between the EtOH and CON groups in both positive and negative ionization modes. Furthermore, the OPLS-DA score plots revealed a remarkable separation of these two groups under both modes (Figure 10). In total, 768 metabolites were up regulated (262 in positive ionization mode and 506 in negative ionization mode), and 1,333 metabolites were down regulated (1,065 in positive ionization mode and 268 in negative ionization mode) in the EtOH group compared to the CON group (VIP > 1 and *p* < 0.05, showed in Figures 11A,B; Supplementary Data S3, S4), and unsupervised clustering heatmap showed in Figures 11C,D. Additionally, most of the significant differential metabolites belong to glycerophospholipids, carboxylic acids and derivatives, fatty acyls, and glycerolipids (Figure 11E), and which were classified as amino acids, peptides and analogs, glycerophosphoethanolamines, glycerophosphocholines, and diradylglycerols in the subclass level (Supplementary Figure S2). The concentration of sphingosine 1-phosphate was increased in the EtOH group, whereas the concentrations of butyric acid, arachidonic acid, L-cysteine, L-tryptophan, serotonin, aldosterone, and farnesyl pyrophosphate were decreased in this group (Supplementary Figure S4). Moreover, the KEGG enrichment analysis of the differential metabolites showed that the lipid metabolism, amino acids metabolism, and carbohydrate metabolism pathways were involved in the AUD process, including sphingolipid metabolism, linoleic acid metabolism, glycerophospholipid metabolism, tryptophan metabolism, and lysine degradation (Figure 11F). The results showed that AUD also altered the serum metabolic profile in rats.

### The correlations of the serum metabolites and gut microbiota

**Figure 12:**
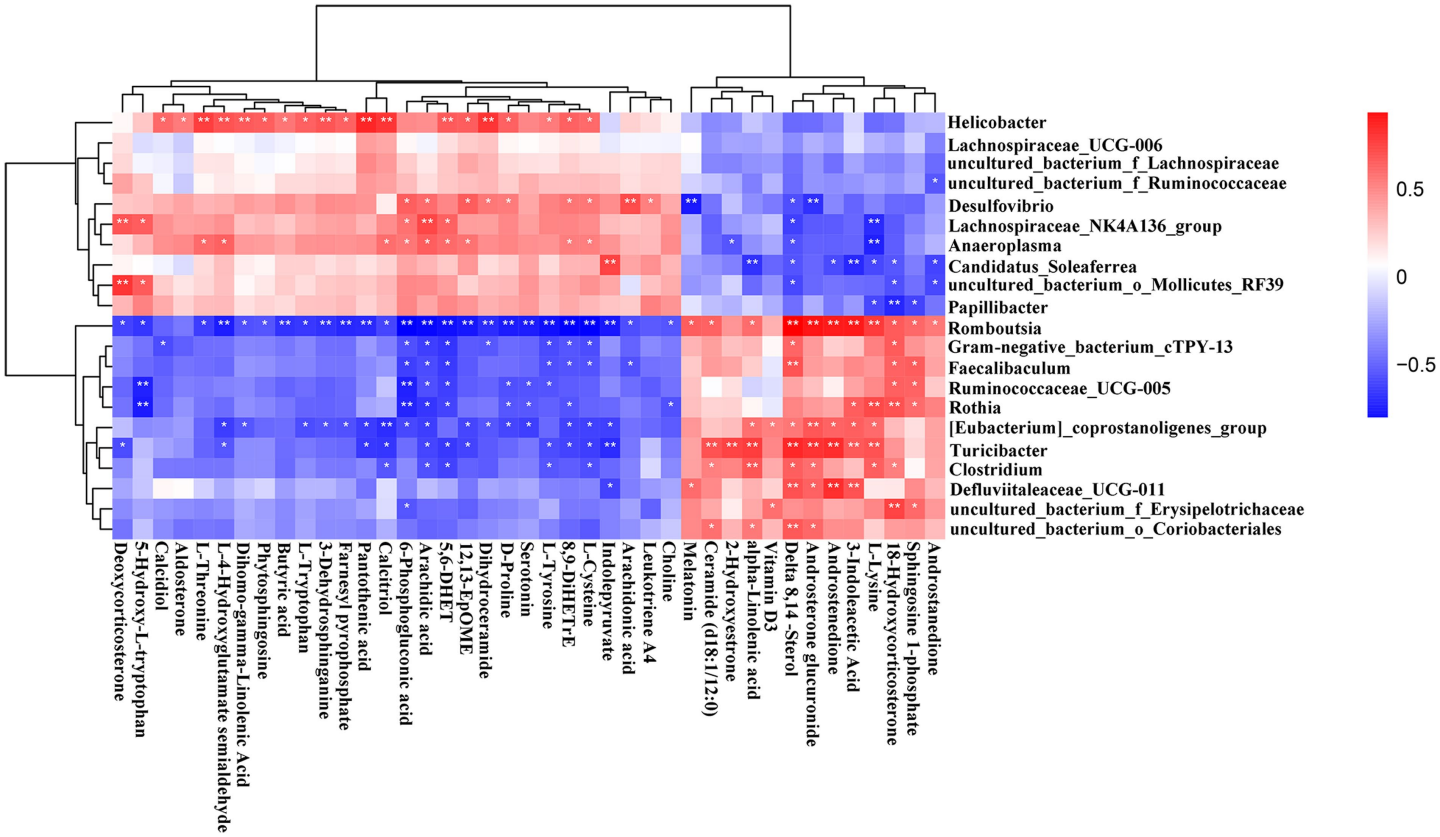
Correlation heatmap of differential microbiota and serum metabolites. Data were calculated by Spearman’s correlation method after mean centering and unit variance scaling. *p < 0.05, **p < 0.01.

The altered serum metabolic profile may reflect the functions of the gut microbiota. Here the functional correlations were explored between the changed microbiota and the top 40 significantly enriched in the KEGG pathway metabolites by Spearman’s correlation analysis (Figure 12). The results showed that *Helicobacter* was positively correlated with L-threonine, L-4-hydroxyglutamate semialdehyde, dihomo-gamma-linolenic acid, 3-dehydrosphinganine, pantothenic acid, calcidiol, 5,6-DHET, and dihydroceramide. Additionally, *Desulfovibrio* was positively correlated with arachidic acid but negatively correlated with androsterone glucuronide and melatonin. Moreover, *Lachnospirace-ae_NK4A136_group* was positively correlated with arachidonic acid and deoxycorticosterone but negatively correlated with L-lysine. The results also showed that the *uncultured_bacterium_o_Mollicutes_RF39* was positively correlated with deoxycorticosterone. *Romboutsia* and *Turicibacter* were positively correlated with delta 8,14-Sterol, androsterone glucuronide, androstenedione, 3-indoleacetic acid, and L-lysine. On the other hand, *Romboutsia* was negatively correlated with L-4-hydroxyglutamate semialdehyde, butyric acid, 3-dehydrosphinganine, farnesyl pyrophosphate, pantothenic acid, 6-phosphogluconic acid, arachidonic acid, 5,6-DHET, 12,13-EpOME, dihydroceramide, D-proline, serotonin, L-tyrosine, 8,9-DiHETrE, L-cysteine, and indolepyruvate.

### Correlations of gut microbiota with the co-regulated metabolites in fecal and serum in AUD rats

**Figure 13:**
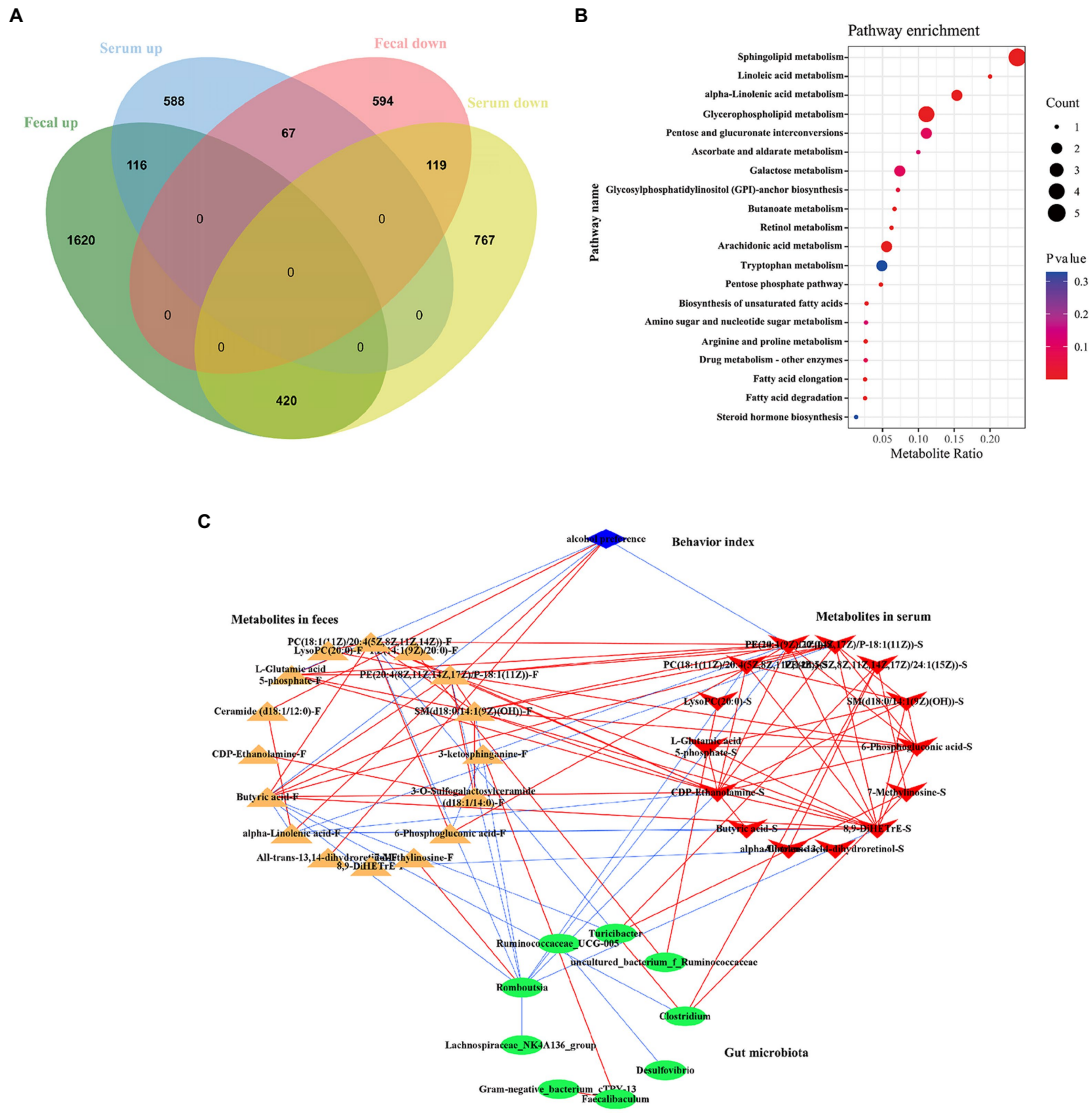
Correlations of gut microbiota with the co-regulated metabolites in feces and serum in AUD rats. The Venn diagram showed the co-regulated metabolites in feces and serum **(A)**. The enrichment pathway of co-regulated metabolites by KEGG analysis **(B)**. Network analysis among the AUD behavior, gut microbiome and co-regulated metabolites **(C)**, the red line represented the positive correlation, the blue line represented negative correlation, |*r*| > 0.8, *p* < 0.05.

The metabolites of feces and serum naturally differ greatly, because of the different metabolic pathways in gut and blood. The blood transports nutrients which absorbed from the digestive tract to the cells and carries away other waste products. So, in feces and serum, there would be some coexisting metabolites, and among these metabolites which were altered by ethanol consumption must play a key role in the pathogenesis of AUD. Here, we used a Venn diagram to screen the co-regulated metabolites in fecal and serum (Figure 13). There are 116 co-up regulated metabolites and 119 co-down regulated metabolites in both feces and serum by ethanol consumption (Figure 13A; Supplementary Data S5). The KEGG enrichment analysis showed that many pathways were altered in AUD rats, such as sphingolipid metabolism, glycerophospholipid metabolism, linolenic acid metabolism, butanoate metabolism, and arachidonic acid metabolism (Figure 13B). We selected the 19 metabolites which were significantly enriched in the KEGG pathway to perform Spearman’s correlation analysis with the changed microbiota (Figure 13C). There are multiple correlations among the AUD behavior, metabolites and gut microbiota. Alcohol preference was positively correlated with ceramide (d18:1/12:0), alpha-linolenic acid, and all-trans-13,14-dihydroretinol in feces, negatively correlated with L-glutamic acid 5-phosphate, butyric acid in feces, and PE(14:1(9Z)/20:0) in serum. *Romboutsia* was negatively correlated with butyric acid, SM(d18:0/14:1(9Z)(OH)), PC(18:1(11Z)/20:4(5Z,8Z,11Z,14Z)), PE(20:4(8Z,11Z,14Z,17Z)/P-18:1(11Z)) in feces, and PE(14:1(9Z)/20:0), 8,9-DiHETrE, CDP-Ethanolamine, PC(18:1(11Z)/20:4(5Z,8Z,11Z,14Z)) in serum. *Turicibacter* and *Clostridium* were negatively correlated with butyric acid.

## Discussion

In this study, the rats that underwent the IA2BC procedure showed a gradual escalation of EtOH intake and increased preference toward EtOH, in addition, in the behavioral tests, the rats exhibited substantial anxiety-like behavior during the withdrawal period. All these phenomena match major symptoms of human AUD listed in the DSM-V, indicating a successful establishment of a rat AUD model in terms of face and construct validity. Moreover, to make a profile of the alteration and correlations of the gut microbiome, fecal, and serum metabolome to further provide a new angle to manage the retractable human AUD besides the neurological perspective, we collected feces and blood from the rats. And, we found the gut microbiota community structure and composition were significantly altered in the EtOH group detected by full length 16S rRNA gene sequencing, and fecal and serum metabolome characteristics were changed in the EtOH group. Finally, multiple correlations among AUD behavior, gut microbiota and the metabolites were also identified. These findings suggest that gut microbiome and serum metabolome may constitute a new pharmacological target for the treatment of human AUD.

Numerous studies have confirmed that gut microbiota is a crucial determinant of health and disease (Dinan and Cryan, 2017; Bajaj, 2019; Pitocco et al., 2020; Witkowski et al., 2020). Accumulating evidence suggests that alcohol consumption can disturb the gut microbiota, increase intestinal permeability and inflammation levels in the gut, and influence behavior in alcohol dependence (Leclercq et al., 2014a,b; Yang et al., 2021). Furthermore, the gut dysbiosis induced by alcohol consumption may promote the development of alcohol addiction (Leclercq et al., 2017). Using the full-length 16S rRNA gene amplicon sequencing, it was found that AUD significantly altered the gut microbiota composition in rats. At the level of phylum taxon, Firmicutes was significantly increased, whereas the Bacteroidetes decreased slightly, leading to a Firmicutes to Bacteroidetes (F/B) ratio significantly increased in the EtOH group than in the CON group. It has been described that Firmicutes and Bacteroidetes are the two main phyla of the gut microbiota (Consortium, 2012). Furthermore, the F/B ratio has been associated with different pathological states, such as inflammatory bowel disease (Stojanov et al., 2020) and type 2 diabetes mellitus (Hung et al., 2021), and is a relevant biomarker of gut dysbiosis in obesity (Indiani et al., 2018) and aging-related processes (Vaiserman et al., 2020). This result is in agreement with prior observations performed by Wang and colleagues, where it has been observed that the F/B ratio increased in the chronic alcohol consumption mice (Wang et al., 2018). Interestingly, Tenericutes, a bacteria phylum lacking a peptidoglycan cell wall, was decreased in the EtOH group, which may be due to the decrease of one of the well-studied gut lineages, *uncultured_bacterium_o_Mollicutes_RF39*, which is rich in H_2_O_2_ catabolism genes and probably produce acetate and hydrogen (Wang et al., 2020). Ethanol metabolism generates reactive oxygen species (ROS) through a cytochrome P450-dependent mechanism (Wu and Cederbaum, 2003). This study found that alcohol reduced the abundance of bacteria that can hydrolyze H_2_O_2_ (one ROS species), leading to a high concentration of ROS in the gut and changes in the microbiome composition. The results also showed that *Lachnospiraceae_NK4A136_group*, members of the Lachnospiraceae, were decreased in the EtOH group compared to the CON group. It has been described that Lachnospiraceae can digest carbohydrates to produce butyrate, which has been considered an anti-inflammatory factor (Flint et al., 2012). In this study, the results showed a decreased level of butyric acid in both fecal and serum metabolites. Some studies revealed that the *Lachnospiraceae_NK4A136_group* decreased in mice with alcohol-induced inflammation (Xia et al., 2021). Some bacteria are increased in the EtOH group at the species level, such as *C. disporicum*, *R. ilealis*, and *T. sanguinis*. For example, *C. disporicum*, a gram-positive saccharolytic bacterium, is known to be an ursodeoxycholic acid producer in the fecal content of rats (Tawthep et al., 2017). Moreover, it is one of the mucin degraders in the human gut, using mucin as the sole carbon and nitrogen source (Raimondi et al., 2021). Mucins are major mucus components that cover the gastrointestinal tract and protect against exogenous and endogenous aggressive agents (Alemao et al., 2021). Therefore, with the increase of *C. disporicum* abundance, gut mucosa degradation increased, promoting gut barrier damage and triggering systemic inflammatory responses. Additionally, *R. ilealis* has been proposed as a potentially harmful bacterium that significantly increases in patients with neurodevelopmental disorders and chemically induced murine colitis model (Bojović et al., 2020; Liu et al., 2022). Moreover, some reports showed that *T. sanguinis* is a strictly anaerobic, gram-positive, pathogenic potential bacterium in the gut and feces of many animals (Cuív et al., 2011; Kim et al., 2021). *T. sanguinis* was capable of serotonin uptake and altered intestinal expression of multiple gene pathways, including those critical for lipid and steroid metabolism (Fung et al., 2019; Hoffman and Margolis, 2020). Our results showed that AUD decreases the potential beneficial bacterium, whereas it increases the pathogenic bacterium that leads to gut dysbiosis.

Gut microbiota has an array of enzymes for digesting the dietary nutrients and regulating the metabolism of various substances in the host, such as short chain fatty acids (SCFAs), organic acids, neuroactive compounds, polyamines, bile acid, choline, and polyphenol (Adak and Khan, 2019). The gut-derived metabolites can act locally in the gut or enter the host’s bloodstream *via* the portal vein (Fan and Pedersen, 2021). Some of the gut-derived bioactive metabolites are associated with regulating host metabolites and metabolic pathways, thereby affecting the fecal or serum metabolome. Under normal physiological conditions, the body’s metabolites and metabolic pathways maintain homeostasis. However, this homeostasis may be disrupted by gut dysbiosis. For example, gut dysbiosis observed in alcoholics could perpetuate and promote addiction through alterations in the metabolism and neuronal pathways (Day and Kumamoto, 2022). In the serum metabolites in AUD patients, the neuroprotective kynurenic acid is decreased, whereas the neurotoxic metabolite quinolinic acid is increased. It has been described that the kynurenic acid / quinolinic acid ratio is positively correlated with the fecal abundance of the genus *Faecalibacterium* (Leclercq et al., 2021). The results showed that the significant differential metabolites in the fecal and serum were similar, belonging to amino acids, peptides, glycerophosphoethanolamines, glycerophosphocholines, and diradylglycerols. Furthermore, the KEGG pathway enrichment analyses of differential metabolites in the fecal and serum showed that sphingolipid metabolism, glycerophospholipid metabolism, steroid hormone biosynthesis, histidine metabolism, tryptophan metabolism, drug metabolism - cytochrome P450, and pentose phosphate pathway were involved in the process of AUD. Sphingolipids are a class of complex lipids that are structural molecules of cell membranes playing a vital role in maintaining membranes’ barrier function and fluidity in eukaryotic cells (Hannun and Obeid, 2008). Several studies suggest that some sphingolipids are bioactive and involved in pathological processes, including cancer, inflammation-associated illnesses, obesity, and neurodegeneration in human and animal models (Gomez-Larrauri et al., 2020; Alaamery et al., 2021). In this study, an upregulation in the sphingolipid metabolism was detected in the fecal and serum of the EtOH group, especially the ceramide and sphingosine-1-phosphate (S1P), which are the best studies bioactive substances in (Hawkins et al., 2020). Furthermore, the gut microbiota can produce sphingolipids to improve the resistance to stress (An et al., 2011), and these sphingolipids can be traced to various tissues throughout the body in mice (Fukami et al., 2010), leading to systemic changes in lipid metabolism (Heaver et al., 2018). In addition to sphingolipid metabolism, two other relevant lipid metabolic pathways, glycerophospholipid metabolism and steroid hormone biosynthesis are also modulated by alcohol consumption. Glycerophospholipids (GPLs) are the main components of biological membranes and are essential for cellular functions. Also, lysophosphatidylcholine (LysoPC) is one of the most prominent lysoglycerophospholipids in the glycerophospholipid metabolism pathway and can be produced by phosphatidylcholine (PC) hydrolysis. It has been described that LysoPC is a type of bioactive metabolite with a high circulating concentration in the body and can trigger immune-related signaling pathways causing immune-related diseases (Ding et al., 2011; Zeng et al., 2017; Tseng et al., 2018). This study found that LysoPC was increased in fecal and serum of the EtOH group and PC was decreased, indicating that alcohol consumption may damage the cell membrane and upregulate the glycerophospholipid metabolism inducing inflammatory responses. The results also revealed that steroid hormone biosynthesis was altered in the EtOH group, characterized by the upregulation of many metabolites such as 18-hydroxycorticosterone, androstenedione, cortisol, and corticosterone. It has been reported that cortisol and corticosterone are the main glucocorticoids produced in the adrenal cortex and strongly affect memory and learning in several animals (Schiffer et al., 2019). Previous studies indicate that corticosterone is involved in alcohol-induced intestinal epithelial dysfunction and alterations in the gut microbiota, playing a crucial role in the stress-induced promotion of alcohol-associated tissue injury at the gut-liver-brain axis (Shukla et al., 2021).

Additionally, in amino acid metabolism, some metabolic pathways were altered in serum compared to the feces in the EtOH group. In the serum, lysine, arginine, proline, glycine, serine, threonine, cysteine, methionine, and tryptophan metabolism pathways were down regulated. Most altered amino acids act as functional amino acids, which regulate critical metabolic and signaling pathways for oxidative stress protection, gut barrier, and immune function. Many studies have proved the key role of arginine, methionine, and cysteine in enhancing immune function through the mTOR activation, NO and glutathione synthesis, H_2_S signaling, and cellular redox state (Li et al., 2007; Wu, 2009). Cysteine and glycine are precursors for synthesizing glutathione, the major antioxidant in cells. Arginine is one of the most versatile amino acids, as it is metabolically interconvertible with proline and can be synthesized into protein, nitric oxide, and creatine (Morris, 2007). Arginine can maintain intestinal integrity and promote the repair of damaged intestinal epithelium by activating mTOR and other kinase-mediated signaling pathways in intestinal epithelial cells (Ban et al., 2004). It has been reported that L-arginine can reverse the anxiolytic and forgetting effects induced by quinpirole (Zarrindast et al., 2021). On the other hand, proline, cysteine, and tryptophan can protect mammalian cells against oxidative stress agents (Krishnan et al., 2008). Additionally, tryptophan is a precursor of serotonin, which is a neurotransmitter that acts as a hormone playing an important role in the regulation of depression, anxiety, and digestive system. Most tryptophan is degraded through the kynurenine pathway metabolism in mammals, and previous studies found that the modulation of the kynurenine pathway is associated with gut microbiota changes, peripheral inflammation, and psychological symptoms in the AUD (Schröcksnadel et al., 2006; Vécsei et al., 2013; Leclercq et al., 2021). Interestingly, in this study, the amino acids described above were downregulated in the EtOH group, indicating that the intestinal permeability, oxidative stress, and immune response induced by alcohol are related to the disorder of amino acid metabolism.

There is also limitation in this study need to be presented. Whether the changes of metabolites in feces and serum are directly caused by alcohol consumption or gut microbiota is not clear. The fecal microbiota transplantation experiment, the relative bacteria strains inoculation test or the key metabolites up/down regulations should be considered in the future research. In conclusion, the altered metabolites in feces and serum are important links between the gut microbiota dysbiosis and alcohol preference in AUD rats, and the altered gut microbiota and metabolites can be potentially new targets for treating AUD.

## Data availability statement

The data presented in the study are deposited in the National Microbiology Data Center (https://nmdc.cn/) repository, accession number is NMDCX0000138.

## Ethics statement

The animal study was reviewed and approved by Animal Ethics Committee of the Qiqihar Medical University.

## Author contributions

CZ, XW, and RZ designed the experiments. XW, LL, CB, and MB performed the animal experiments. XW and LL conducted gut microbiota analysis and wrote the draft of the manuscript. XW, LL, JZ, HG, and HY conducted the metabolites analysis. All authors contributed to the article and approved the submitted version.

## Funding

This research was funded by Academy of Medical Sciences in Qiqihar Medical University, grant number: QMSI2017B-13, QMSI2017B-06, and 2021-ZDPY-003; by Qiqihar Science and Technology Plan Joint Guidance Project, grant number: LSFGG-2022055, and LHYD-2021014, by Health commission of Heilongjiang Province, grant number: 20210101060181; Heilongjiang undergraduate Innovation and Entrepreneurship Project, grant number: 202111230016.

## Conflict of interest

The authors declare that the research was conducted in the absence of any commercial or financial relationships that could be construed as a potential conflict of interest.

## Publisher’s note

All claims expressed in this article are solely those of the authors and do not necessarily represent those of their affiliated organizations, or those of the publisher, the editors and the reviewers. Any product that may be evaluated in this article, or claim that may be made by its manufacturer, is not guaranteed or endorsed by the publisher.

## Supplementary material

The Supplementary material for this article can be found online at: https://www.frontiersin.org/articles/10.3389/fmicb.2022.1068825/full#supplementary-material

## Footnotes

^1^ https://www.metaboanalyst.ca/MetaboAnalyst/home.xhtml
