# Supplementary material for: Alterations and correlations of gut microbiota, fecal, and serum metabolome characteristics in a rat model of alcohol use disorder
Source: Front Microbiol. 2023 Jan 4;13:1068825. doi: 10.3389/fmicb.2022.1068825 (PMC9846065; doi:10.3389/fmicb.2022.1068825)
Supplement: Supplementary file 2 [file Data_Sheet_1.PDF]

Supplementary Material

1 Supplementary Data

Supplementary Data 1 In positive ionization mode, a total of 2076 metabolites were up regulated and 288 metabolites were down regulated in fecal samples.

Supplementary Data 2 In negative ionization mode, a total of 80 metabolites were up regulated and 492 metabolites were down regulated in fecal samples.

Supplementary Data 3 In positive ionization mode, a total of 262 metabolites were up regulated and 1065 metabolites were down regulated in serum samples.

Supplementary Data 4 In negative ionization mode, a total of 506 metabolites were up regulated and 268 metabolites were down regulated in serum samples.

Supplementary Data 5 The co-regulated metabolites in both fecal and serum.

2 Supplementary Figures and Tables

2.1 Supplementary Tables

Supplementary Table 1. Sequencing data quality of full length 16S rRNA gene sequencing

	Sample ID	Raw CCS	Clean CCS	Effective CCS	AvgLen(bp)	Effective(%)
CON	C1	10,312	9,192	9,173	1,457	88.95
	C2	13,039	11,401	11,365	1,455	87.16
	C3	13,011	11,619	11,616	1,459	89.28
	C4	12,942	11,687	11,683	1,456	90.27
	C5	12,976	11,700	11,694	1,460	90.12
	C6	12,834	11,752	11,741	1,459	91.48
	C7	12,999	11,782	11,777	1,459	90.6
	C8	12,969	11,677	11,669	1,462	89.98
EtOH	E1	12,983	11,752	11,738	1,455	90.41
	E2	13,021	11,783	11,779	1,454	90.46
	E3	11,745	10,616	10,612	1,452	90.35
	E4	11,375	10,250	10,242	1,451	90.04
	E5	10,680	9,698	9,695	1,455	90.78
	E6	11,886	10,772	10,760	1,451	90.53
	E7	12,880	11,719	11,712	1,453	90.93
	E8	10,196	9,240	9,238	1,453	90.6

2.2 Supplementary Figures

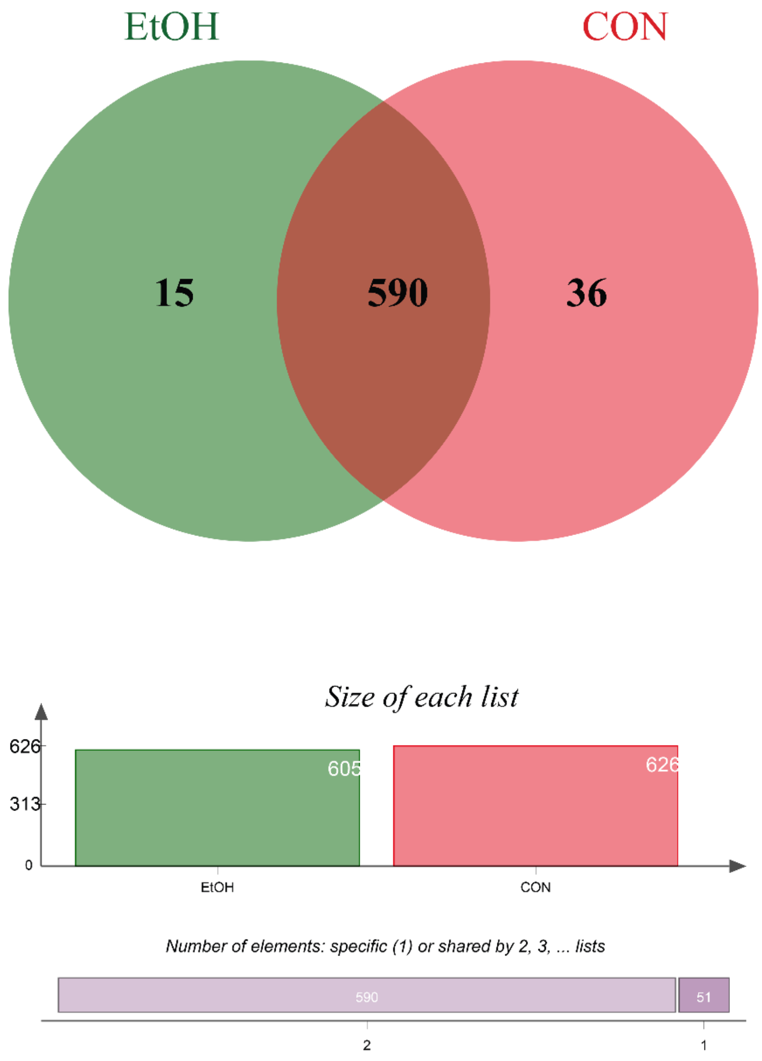

Supplementary Figure 1 The venn diagram for OTUs of gut microbiota in EtOH and CON group.

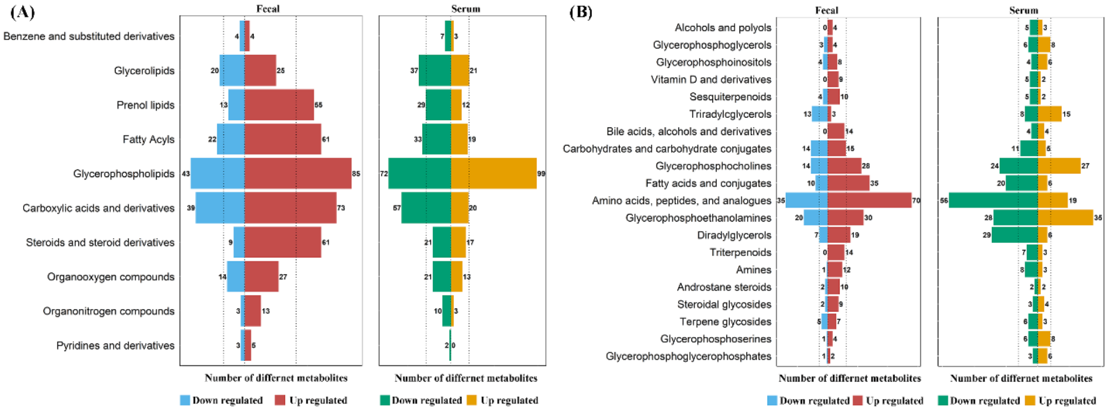

Supplementary Figure 2 The number of differential metabolites were annotated and classified based on the HMDB database.

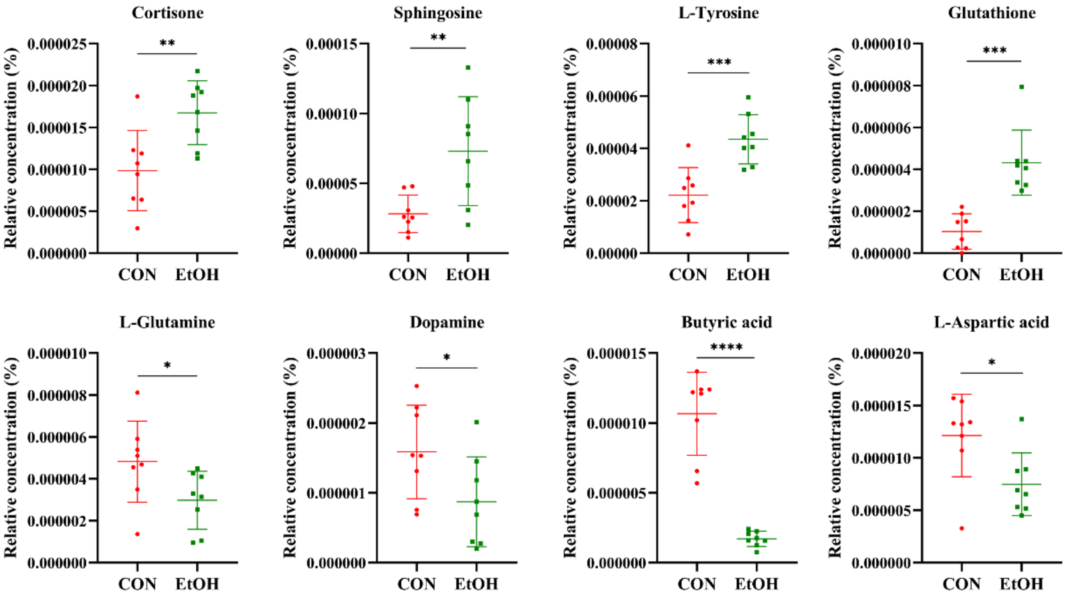

Supplementary Figure 3. The relative concentrations of the altered metabolites in the fecal samples, * $p < 0.05$, ** $p < 0.01$, *** $p < 0.001$.

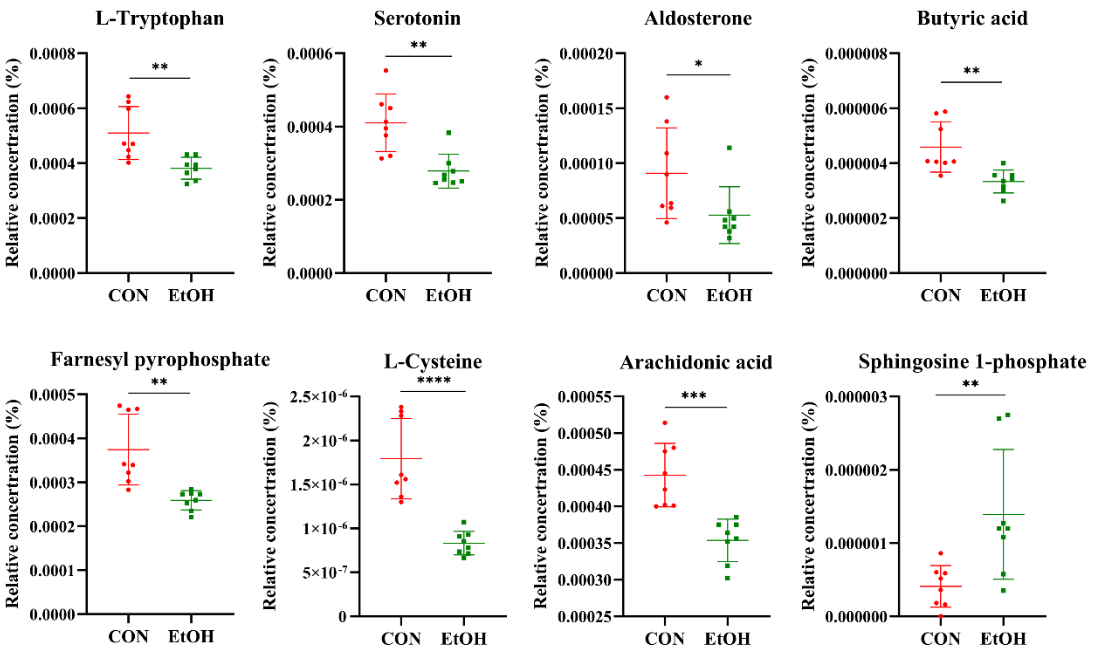

Supplementary Figure 4. The relative concentrations of the altered metabolites in the serum samples, * $p < 0.05$, ** $p < 0.01$, *** $p < 0.001$, **** $p < 0.0001$.
